# Prescribing of clotrimazole-betamethasone dipropionate, a topical combination corticosteroid-antifungal product, for Medicare part D beneficiaries, United States, 2016–2022

**DOI:** 10.1017/ash.2024.435

**Published:** 2024-10-17

**Authors:** Dustin W. Currie, Avrom S. Caplan, Kaitlin Benedict, Kelly M. Hatfield, Dallas J. Smith, Shari R. Lipner, Jeremy A.W. Gold

**Affiliations:** 1 Centers for Disease Control and Prevention, Atlanta, GA, USA; 2 The Ronald O. Perelman Department of Dermatology, NYU Grossman School of Medicine, New York, NY, USA; 3 Israel Englander Department of Dermatology, Weill Cornell Medicine, New York, NY, USA

## Abstract

During 2016–2022, Medicare part D beneficiaries filled 8,674,460 clotrimazole-betamethasone dipropionate prescriptions. Annual rates were stable (30.9 prescriptions/1,000 beneficiary-years in 2022, enough for one in every 33 beneficiaries). Diagnostic testing was infrequent, particularly among internal medicine, family medicine, and general practitioners, suggesting potential opportunities to improve diagnostic and prescribing practices.

## Introduction

Clotrimazole-betamethasone dipropionate (Lotrisone®), available as a cream or lotion, is the most frequently prescribed topical antifungal-corticosteroid medication in the United States.^1,2^ Fungal skin infections, such as dermatophytosis (ie, ringworm and tinea) and cutaneous candidiasis, are common outpatient diagnoses with an estimated worldwide lifetime prevalence exceeding 20%.^3^ Visual inspection does not reliably differentiate fungal skin infections, which are often treated with topical antifungals, from similar-appearing nonfungal inflammatory skin conditions (eg, eczema and psoriasis) that may benefit from anti-inflammatory treatment using topical corticosteroids.^4,5^ Diagnostic testing (eg, direct microscopy) can establish a fungal diagnosis and lead to appropriate therapy.^5^

When skin condition etiology is uncertain, clinicians might attempt to empirically treat both fungal infections and nonfungal inflammatory conditions by prescribing topical antifungal-corticosteroid combination products.^1,6^ However, prescribing topical antifungal-corticosteroid combination products is discouraged because they are potentially less effective and more costly than antifungal monotherapy; can cause adverse effects including skin atrophy and hypothalamic–pituitary–adrenal axis suppression; and are hypothesized to drive the emergence and spread of antifungal-resistant skin infections.^1,2,7^ In the United States, severe, antimicrobial-resistant fungal skin infections have recently emerged, warranting increased attention to judicious topical antifungal-corticosteroid prescribing.^2,8,9^

In 2021, Medicare part D beneficiaries filled approximately 1 million clotrimazole-betamethasone dipropionate prescriptions, which is concerning because older adults are particularly susceptible to the adverse skin and systemic effects.^2,10^ Data on trends in clotrimazole-betamethasone dipropionate use in adults are limited,^1,7^ and understanding clotrimazole-betamethasone dipropionate-related diagnostic testing and prescribing, particularly when potentially inappropriate (eg, for intravaginal use or non-fungal conditions), could help identify opportunities to improve clinical practice.^2^ Clotrimazole-betamethasone use in older adults is of particular concern as superficial fungal skin infections are particularly common in this population and older adults may be more susceptible to adverse effects from high potency topical steroids.^2^ Therefore, we analyzed clotrimazole-betamethasone dipropionate prescriptions among Medicare part D beneficiaries.

## Methods

Using Medicare Part D event file data (https://resdac.org/#find-cms-data-file) for this cross-sectional analysis, we identified clotrimazole-betamethasone dipropionate prescriptions filled during 2016–2022 by beneficiaries aged ≥65 years. We tabulated total and yearly prescription numbers and described annual rates per 1,000 beneficiary-years with Medicare Part D coverage by age group (65–74, 75–84, 85–94, and ≥95 years), sex, and U.S. Census region (https://www.census.gov/programs-surveys/economic-census/guidance-geographies/levels.html). We calculated total and yearly numbers of prescriptions by prescriber type (internal medicine physicians [IMs], family medicine and general practice physicians [FM/GPs], nurse practitioners and physician assistants [NP/PAs], obstetrician-gynecologists, and other prescriber types).

We further characterized prescribing practices among beneficiaries prescribed clotrimazole-betamethasone dipropionate during 2022 who had Medicare Parts A and B fee-for-service and Part D prescription drug coverage for at least 11 months of the year using the first filled prescription in the calendar year (index prescription). For beneficiaries with a Part D event file for a filled clotrimazole-betamethasone dipropionate prescription, we examined diagnostic testing (using current procedural terminology [CPT] codes) and select diagnoses (using International Classification of Diseases, 10th Revision [ICD-10-CM] codes) on Medicare carrier and outpatient claims on or within the 28 days before or after their index prescription. The frequency of relevant procedures and diagnoses was stratified by prescriber type of the index prescription. Descriptive analyses were performed using SAS Studio.

## Results

**Figure 1.**
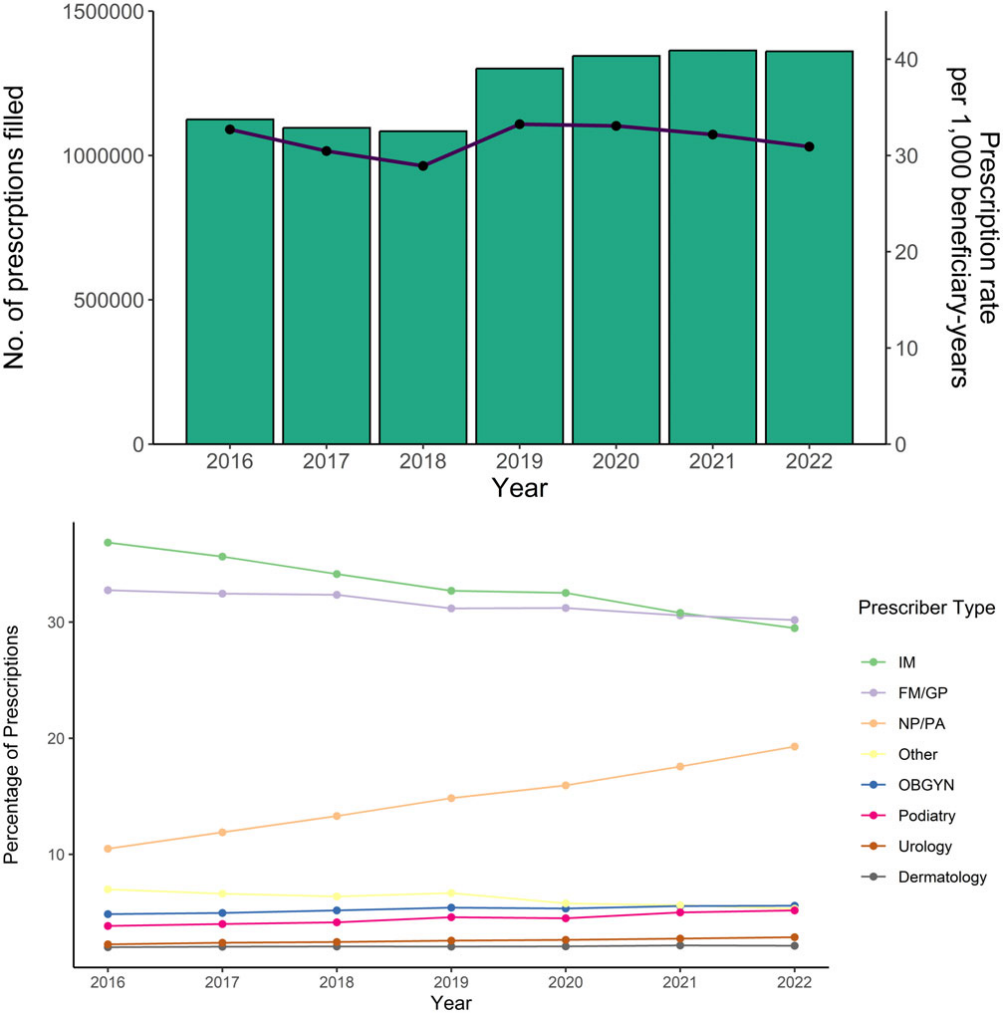
Annual numbers and rates of clotrimazole-betamethasone prescriptions (a) and (b) percentage distribution by presciber type among Medicare Part D Beneficiaries, United States, 2016–2022^a^. IM, internal medicine physician; FM/GP, family medicine physician/general practitioner; NP/PA, physician assistant/nurse practitioner; OB-GYN, obstetrician-gynecologist. ^a^ Panel B excludes 0 prescriptions and 14 beneficiary-years with unknown gender. Panel C excludes 797,582 prescriptions and 3,611,808 beneficiary-years in U.S. territories.

During 2016–2022, 8,674,460 clotrimazole-betamethasone dipropionate prescriptions were filled, with annual prescription rates generally stable overall (Figure 1). Rates were stable across age groups, sexes, and regions (Supplementary Data). In 2022, 1,360,703 prescriptions were filled among 44,011,116 beneficiaries (rate: 30.9/1,000 beneficiary-years); rates were highest among older beneficiaries (43.2 for ≥95 years vs. 26.9 for 65–74 years) and in the Northeast (40.3) compared with the South (33.7), Midwest (20.5), and West (18.0). Rates were similar for men (30.0) and women (31.6).

Overall, most clotrimazole-betamethasone dipropionate prescriptions were written by IMs (32.6%) or FP/GPs (31.1%), followed by NP/PAs (14.9%) and obstetrician-gynecologists (5.2%) (Figure 1). From 2016 to 2022, a decreasing percentage of prescriptions were from IMs (35.8% to 29.3%), an increasing percentage were from NP/PAs (10.2% to 19.2%), and the percentage from each other group changed by <2.0 percentage points.

In 2022, among beneficiaries who filled a clotrimazole-betamethasone dipropionate prescription and met sub-analysis inclusion criteria (n = 269,558), 24,440 (9.1%) received a diagnostic test (Table 1). Testing rates were highest for beneficiaries prescribed clotrimazole-betamethasone dipropionate by an obstetrician-gynecologist (19.6%), followed by other prescriber types (11.6%), NP/PAs (10.9%), IMs (5.9%), and FM/GPs (5.7%). Across specialties, <40% of beneficiaries prescribed clotrimazole-betamethasone dipropionate had a fungal diagnosis, with the lowest percentage among those prescribed by obstetrician-gynecologists (24.8%). Among beneficiaries without a fungal diagnosis (n = 185,660, 68.9%), “dermatitis and eczema” (12.5%) and “rash and other nonspecific skin eruption” (10.0%) were the most common identified diagnoses across all prescriber types, except for beneficiaries prescribed by obstetrician-gynecologists, where genital conditions were most common (20.3%, of which 64.3% were acute vaginitis).

**Table 1.** Diagnostic testing, diagnoses, and repeated prescriptions for Medicare beneficiaries prescribed clotrimazole-betamethasone dipropionate (n = 269,558), by prescriber type — United States, 2022^a^

Characteristic	All (n = 269558)	IM (n = 71887)	FM/GP (n = 67274)	NP/PA (n = 56199)	OB-GYN (n = 21354)	Other^d^ (n = 51786)
**Diagnostic testing** ^b^	**24440 (9.1)**	**4221 (5.9)**	**3810 (5.7)**	**6117 (10.9)**	**4188 (19.6)**	**6014 (11.6)**
Fungal culture	978 (4.0)	105 (2.5)	86 (2.3)	297 (4.9)	130 (3.1)	360 (6.0)
Direct microscopy	1663 (6.8)	128 (3.0)	175 (4.6)	538 (8.8)	540 (12.9)	280 (4.7)
Susceptibility testing	9263 (37.9)	2039 (48.3)	1691 (44.4)	2381 (38.9)	1297 (31.0)	1829 (30.4)
Skin biopsy	8780 (35.9)	1590 (37.7)	1400 (36.7)	1996 (32.6)	635 (15.2)	3115 (51.8)
Polymerase chain reaction	6356 (26.0)	640 (15.2)	673 (17.7)	1709 (27.9)	2239 (53.5)	1071 (17.8)
**Fungal diagnosis present**	**83898 (31.1)**	**19346 (26.9)**	**19889 (29.6)**	**18522 (33.0)**	**5288 (24.8)**	**20593 (39.8)**
Dermatophytosis	61612 (73.4)	14834 (76.7)	14978 (75.3)	11694 (63.1)	1974 (37.3)	17938 (87.1)
Candidiasis	21495 (25.6)	3955 (20.4)	4696 (23.6)	6826 (36.9)	3527 (66.7)	2432 (11.8)
Other superficial mycoses	4608 (5.5)	1453 (7.5)	1003 (5.0)	1197 (6.5)	131 (2.5)	805 (3.9)
Unspecified mycoses	534 (0.6)	177 (0.9)	123 (0.6)	140 (0.8)	22 (0.4)	71 (0.3)
**Fungal diagnosis absent**	**185660 (68.9)**	**52541 (73.1)**	**47385 (70.4)**	**37677 (67.0)**	**16066 (75.2)**	**31193 (60.2)**
Dermatitis and eczema^c^	23292 (12.5)	5320 (10.1)	6287 (13.3)	5985 (15.9)	825 (5.1)	4767 (15.3)
Genital conditions^c^	10332 (5.6)	781 (1.5)	931 (2.0)	2780 (7.4)	3259 (20.3)	2550 (8.2)
Acute vaginitis^c^	4515 (43.7)	363 (46.5)	403 (43.3)	1320 (47.5)	2095 (64.3)	319 (12.5)
Rash and other nonspecific skin eruption^c^	18651 (10.0)	5775 (11.0)	5413 (11.4)	5185 (13.8)	476 (3.0)	1752 (5.6)
Other local infections of skin and subcutaneous tissue^c^	8887 (4.8)	2382 (4.5)	1965 (4.1)	2077 (5.5)	405 (2.5)	2024 (6.5)
**Number of prescriptions filled during 2022**						
1	181540 (67.3)	45594 (63.4)	44447 (66.1)	39510 (70.3)	15272 (71.5)	36008 (69.5)
2	48538 (18.0)	13410 (18.7)	12375 (18.4)	9588 (17.1)	3866 (18.1)	9114 (17.6)
3	17739 (6.6)	5399 (7.5)	4567 (6.8)	3345 (6.0)	1130 (5.3)	3228 (6.2)
≥4	21741 (8.1)	7485 (10.4)	5885 (8.8)	3754 (6.7)	1086 (5.1)	3437 (6.6)

IM, internal medicine physician; FM/GP, family medicine physician/general practitioner; NP/PA, nurse practitioner/physician assistant, OB-GYN, obstetrician-gynecologist.

a Data shown as no. (% column). Beneficiaries could have >1 diagnostic test and could receive >1 diagnosis. Columns may not sum to 100% because 1,058 beneficiaries were missing prescriber type. Analyses limited to first CBM prescription for each beneficiary within the calendar year.

b Diagnostic testing could not be linked with certainty to the clotrimazole-betamethasone dipropionate prescription or to a specific diagnosis code.

c Includes only patients without a fungal diagnosis.

d The most common ‘other’ prescriber types include: Podiatry (n = 14,682; 5.4%), Urology (n = 9864; 3.7%), Dermatology (n = 9828; 3.6%), and Otolaryngology (n = 4656; 1.7%).

Overall, 33.7% of beneficiaries had ≥1 additional clotrimazole-betamethasone dipropionate prescription after their initial 2022 prescription, without substantial variation by prescriber type.

## Discussion

Our analysis found that clotrimazole-betamethasone dipropionate prescribing rates for Medicare part D beneficiaries remained stable during 2016–2022, with 1.4 million prescriptions filled during 2022, representing enough to provide one prescription for every 33 beneficiaries. Across specialties, and consistent with past studies, most patients did not receive a fungal infection diagnosis or have diagnostic testing,^6^ prescribing for acute vaginitis was observed, and more than one-third of patients were prescribed clotrimazole-betamethasone dipropionate at least twice, suggesting overuse and potentially inappropriate prescribing. The finding that clotrimazole-betamethasone dipropionate prescribing volume was highest by GPs and FP/IMs and rising for NP/PAs suggests that these groups could be prioritized in efforts to educate prescribers about the potential for adverse effects and resistance selection pressure.^2^

Clotrimazole-betamethasone dipropionate prescribing rates were highest among the oldest beneficiaries, which is concerning because older adults are particularly susceptible to corticosteroid-related adverse effects.^10^ That clotrimazole-betamethasone dipropionate prescribing rates were highest in the Northeast is consistent with previous studies of topical antifungal prescribing patterns, although reasons for this finding are unclear.^2,6^ Efforts to improve prescribing practices could prioritize education for prescriber types who frequently prescribe clotrimazole-betamethasone dipropionate; attempting to understand reasons for frequent prescribing; addressing barriers to diagnostic testing (eg, limited time, regulatory restrictions); and suggesting alternatives to topical antifungal-corticosteroid products such as antifungal monotherapy (ideally, after confirming fungal infections using diagnostic testing), and referral to dermatologists in instances of diagnostic uncertainty.^2,6^

This study had several notable limitations. ICD-10-CM and CPT codes might be subject to disease misclassification, undercoding for conditions or procedures with low reimbursement rates, and imprecise use. It is possible some prescribers would use the term dermatitis to describe suspected fungal infections. Furthermore, diagnostic testing could not be linked with certainty to the prescription or to a specific diagnosis code. Finally, the data set may not be representative of all U.S. adults aged ≥65 years, particularly those without Medicare prescription drug coverage, and information on diagnoses and testing practices were only available for a subset of patients. Nonetheless, we highlight potential opportunities to improve prescribing practices for treating suspected fungal skin infections.

## Supporting information

Currie et al. supplementary material Currie et al. supplementary material

## Supplementary material

For supplementary material accompanying this paper visit http://doi.org/10.1017/ash.2024.435. click here to view supplementary material

## Note

This activity was reviewed by CDC and was conducted consistent with applicable federal law and CDC policy (eg, 45 C.F.R. part 46; 21 C.F.R. part 56; 42 U.S.C. §241(d), 5 U.S.C. §552a, 44 U.S.C. §3501 et seq). Use of trade names and commercial sources is for identification only and does not imply endorsement by the U.S. Department of Health and Human Services or the U.S. Centers for Disease Control and Prevention.
