# Supplementary material for: Prescribing of clotrimazole-betamethasone dipropionate, a topical combination corticosteroid-antifungal product, for Medicare part D beneficiaries, United States, 2016–2022
Source: Antimicrob Steward Healthc Epidemiol. 2024 Oct 17;4(1):e174. doi: 10.1017/ash.2024.435 (PMC11500261; doi:10.1017/ash.2024.435)
Supplement: Currie et al. supplementary material [file S2732494X24004352sup001.docx]

| **Supplementary Table.** International Classification of Diseases, Tenth Revision, Clinical Modification (ICD-10-CM) and Current Procedural Terminology (CPT) codes used to identify features of interest | |
| --- | --- |
| **ICD-10-CM codes** | **Diagnosis** |
| **B35** | Dermatophytosis |
| **B37** | Candidiasis |
| **B36** | Other superficial mycoses |
| **B49** | Unspecified mycoses |
| **L20–L30** | Dermatitis and eczema |
| **N47.8, N48.1, N48.9, N76.0, N76.2** | Genital conditions |
| **N76.0** | Acute vaginitis |
| **R21** | Rash and other nonspecific skin eruption |
| **L00–L08** | Other local infections of skin and subcutaneous tissue |
| **CPT codes** | **Diagnostic testing** |
| **87101, 87102, 87106, 87107** | Fungal culture |
| **87210, 87220, 87206** | Direct microscopy |
| **87186** | Susceptibility testing |
| **11100, 11102, 11103, 11104, 11105, 11106, 11107** | Skin biopsy |
| **87481, 87798, 87800, 87801** | Polymerase chain reaction |

**Supplementary Figure.** Annual rates (per 1,000 beneficiary-years) of clotrimazole-betamethasone dipropionate prescriptions by age group (A), sex (B), and U.S. census region (C) among Medicare part D beneficiaries, United States, 2016-2022^a^

**
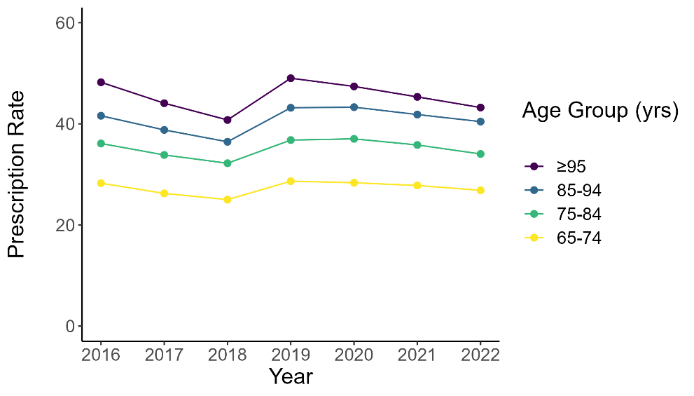

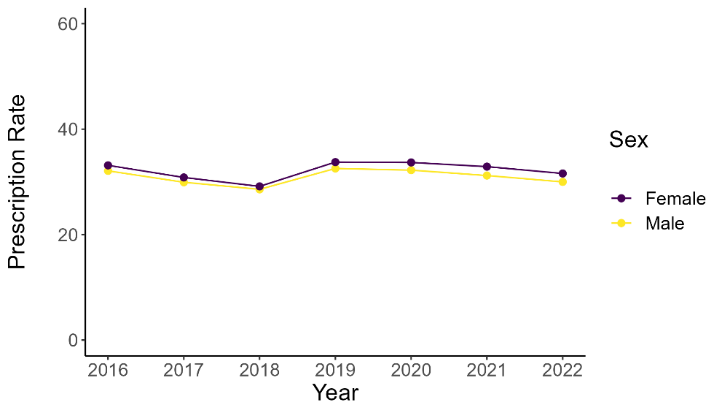
A B**

**
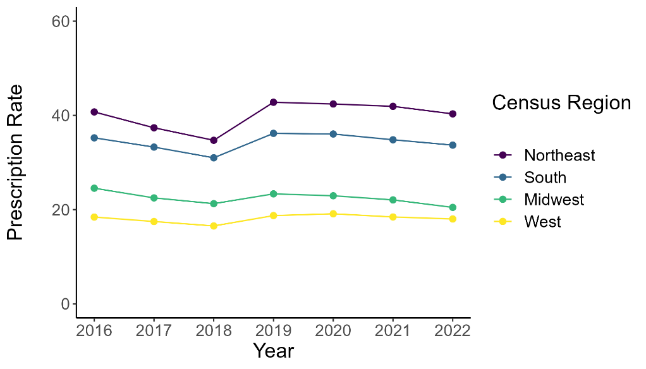
C**

^a^ Panel B excludes 0 prescriptions and 14 beneficiary-years with unknown gender. Panel C excludes 797,582 prescriptions and 3,611,808 beneficiary-years in U.S. territories.
